# Associations among knowledge, attitudes, and practices toward palliative care consultation service in healthcare staffs: A cross-sectional study

**DOI:** 10.1371/journal.pone.0223754

**Published:** 2019-10-11

**Authors:** Li-Chun Huang, Ho-Jui Tung, Pei-Chao Lin

**Affiliations:** 1 Department of Nursing, Zuoying Branch of Kaohsiung Armed Forces General Hospital, Kaohsiung, Taiwan; 2 Department of Healthcare Administration, Asia University, Taichung, Taiwan; 3 Department of Health Policy and Community Health, Jiann-Ping Hsu College of Public Health, Georgia Southern University, Georgia, United States of America; 4 School of Nursing, College of Nursing, Kaohsiung Medical University, Kaohsiung, Taiwan; 5 Department of Medical Research, Kaohsiung Medical University Hospital, Kaohsiung Medical University, Kaohsiung, Taiwan; University of Auckland, NEW ZEALAND

## Abstract

**Background:**

The palliative care consultation service (PCCS) of the National Health Insurance payments has been promoted in Taiwan since 2011, although few studies have been conducted on healthcare staffs’ knowledge, attitudes, and practices regarding PCCS in Taiwan; consequently, the main objective of this study was to explore any correlations regarding the above by cross-sectional design using convenience sampling.

**Methods:**

A total of 210 healthcare staff members were enrolled from a regional hospital from June 1, 2018, to September 30, 2018. Questionnaire items on the Palliative Care Consultation Service Inventory (KAP-PCCSI) were used to measure healthcare staff’s knowledge, attitudes, and practices of PCCS. The collected data were analyzed by using descriptive statistics, independent samples *t-*test, Pearson’s correlation coefficient analysis, and multiple linear regression analysis.

**Results:**

The results revealed that the mean scores for knowledge of and attitudes of KAP-PCCSI were 58.7 ± 8.9 (perfect score: 75) and 42.7 ± 4.7 (perfect score: 50) respectively, while the mean score for practices of KAP-PCCSI was 36.3 ± 8.1 (perfect score: 50); moreover, the healthcare staff’s knowledge and attitudes were positively correlated with their practices (*p* < 0.01). The results also showed that knowledge, attitudes, experience of having a family member(s) or friend(s) passing away, and being a medical personnel constituted the major predictors of practices (*p* < 0.001). These factors explained 43.2% of the overall variance for practices of KAP-PCCSI.

**Conclusions:**

The findings can help healthcare staff understand factors influencing practices of KAP-PCCSI and can serve as a reference for the development of strategies for palliative care education and training while improving the care quality of patients undergoing such palliative care with terminal life considerations in the hospitals, thereby fulfilling the goal of achieving holistic care.

## Introduction

The World Health Organization defines palliative care as an approach that improves the quality of life of patients and their families facing the problem associated with life-threatening illness, through the prevention and relief of suffering by means of early identification and impeccable assessment and treatment of pain and other problems including the physical, psychosocial and spiritual dimensions [1]. It focuses on optimizing the quality of life of patients and their families who have serious and complex illnesses [2].

In Taiwan, cancer has been a leading cause of death in Taiwan for three decades. According to the 2017 statistics provided by Taiwan’s Ministry of Health and Welfare, the mortality rate associated with malignant tumors was 203.9 per 100,000 population, representing an increase of 28 per 100,000 population relative to the rate in 2007 (ie, 175.9 per 100,000 population) [3]. The Ministry of Health and Welfare enacted the Hospice Palliative Care Act in Taiwan in 2000, while the newest amended version is in 2013. The Hospice Palliative Care Act is specifically stipulated to respect the will of terminally ill patients on medical treatment and protect their rights. In addition, the Hospice Palliative Care Act is defined as “hospice palliative care refers to the mitigatory and supportive medical care given to relieve terminal illness patients from and remove physical, mental and spiritual pain, to improve their quality of life.” [4]. The utilization of hospice palliative care was further facilitated by the Taiwanese National Health Insurance in 2000. The Ministry of Health and Welfare has comprehensively promoted the concept of palliative care to increase the quality of life of patients with terminal illness, enabling terminally ill patients to die with dignity. The palliative care consultation service (PCCS) is established to be the advocate of such patients in acute hospitals. The palliative care consultation service (PCCS) encourages collaboration between palliative care team members and medical team members in order to enable the provision of barrier-free palliative care to patients with terminal illness in Taiwan [5], and is comprised of physicians, nurse specialists, social workers, psychologists, pharmacists, dietitians, religious workers, and nursing staff. The benefits of PCCS in Taiwan have indicated that positive care experience indices include satisfaction with treatment of dignity and respect, and comfort-focused treatment goals [2, 6–8].

In other countries, numerous studies have indicated that palliative care consultation services (PCCS) can effectively provide symptomatic treatment, shorten the length of hospital stay, reduce medical costs, increase the quality of the patient’s end-of-life, and improve their satisfaction and that of their family [8–11]. The PCCS provides various options for the care of terminal patients, including physical, psychosocial and spiritual issues. In addition to improving the well-being of patients with terminal illness and their family, the PCCS team provides palliative care advice to primary health professionals according to the patient's condition [2, 7, 8, 12].

Several studies have investigated nursing staff members’ knowledge, attitudes, and practices regarding PCCS in cancer wards for patients with terminal cancer [13]. One study regarding PCCS used purposive sampling to recruit four homogenous focus groups from a large medical center in Los Angeles, with two of the focus groups being comprised of physicians with the remaining two comprised of nurses [14]. Structured interviews were conducted to explore their attitudes toward PCCS, and it was reported that all groups had positive attitudes toward PCCS overall, although some nurses expressed inadequate knowledge as one of the barriers to implementing PCCS [14].

However, whether conventional healthcare staffs understand the definition of PCCS, Hospice Palliative Act, and palliative care purposes needs to be investigated; additionally, exploration of their knowledge of, attitudes toward, and practices of symptom management, nursing care, and communication with patients and their family members with terminal illness, as well as their willingness to accept PCCS in the future is also required.

Few studies have been conducted on these dimensions for healthcare members regarding PCCS in Taiwan, so the main study objective was to explore any correlations between them toward palliative care consultation service, with the aim of providing a reference for education on PCCS-related concepts and clinical practices to enable further provision of high-quality care to patients with terminal illness.

## Materials and methods

### Study design and population

This research was a correlational study with a cross-sectional design. The participants in this study were conveniently sampled from the intensive care unit, internal medicine and surgery general ward, and obstetrics and pediatrics wards of a regional teaching hospital in southern Taiwan in 2018. The inclusion criteria for the study were as follows: (a) being at least 20 years of age, (b) healthcare members working in the ward or intensive care unit (ICU), and (c) able to speak Mandarin or Taiwanese. The exclusion criteria were being unable to provide informed consent or having cognitive impairment. The researcher recruited participants by explaining the objectives and methods of the study with them completing written anonymous and confidential informed consent forms at recruitment. The researcher distributed 230 questionnaires and collected 210 valid responses, signifying a 91.3% valid response rate. Participants were categorized according to their specific occupations, with numbers comprising 39 physicians and nurse practitioners, 140 nurses, and 31 other medical personnel (namely 1 psychologist, 2 social workers, 2 dietitians, 25 pharmacists, and 1 religious worker).

### Questionnaire

We collected data through a structured questionnaire. Participants spent around 15–20 min to fill out the questionnaires. The questionnaire included relevant items from validated questionnaires, including the sociodemographic characteristics of the relative and the knowledge, attitude, and practice regarding the palliative care consultation service inventory (KAP-PCCSI) [15].

The KAP-PCCSI is a self-report questionnaire including three parts: knowledge (15 items), attitude (10 items), and practice (10 items). The three parts are all scored using a 5-point Likert-type scale ranging from 1 to 5. The responses with higher scores indicate better knowledge in PCCS (score ranges from 15 to 75) and more favorable attitudes (score ranges from 10 to 50), practice toward PCCS (score ranges from 10 to 50), except for items 9 and 10 of the attitudes towards PCCS being negatively worded [15].

The knowledge towards PCCS concerns concept, purpose, and responsibility of PCCS, the criteria and methods for applying consultations, and knowing PCCS team members. The attitude towards PCCSI concerns items of healthcare members’ attitudes including the view and value of PCCS, and whether agreeing with the PCCS will help terminal patient treatment, comfort care, quality of life, and family members. The practice toward PCCSI concerns healthcare members’ practices of PCCS that included clinical practice behaviors; for example, after the PCCS, whether the healthcare members would provide comfortable nursing or guidance for patients and family for good death preparation, willingness for autonomous learning and training about PCCS or providing active assistance in referral to the terminal patients to receive PCCS, or share the experience and reflection about PCCS.

The content validity index (CVI) was 0.97 and the Cronbach's alpha scores of K-PCCSI, A-PCCSI, and P-PCCSI was 0.90, 0.86, and 0.93 [15]. Furthermore, content validity was evaluated by five experts: a professor, an associate professor, a physician, a registered nurse and a social worker all working within the field of palliative care in this study. The experts evaluated items of KAP-PCCSI using a 5-point rating scale on relevance, accuracy, and applicability. The CVI across the items was 0.96. The Cronbach alphas of the KAP-PCCSI in this study were 0.92, 0.88, and 0.96 for the 210 healthcare team members respectively.

### Statistical analysis

We used the statistical software package SPSS for Windows, version 20.0 (IBM Corporation, Armonk, NY, USA) to analyze the collected data. Specifically, we described the data using descriptive statistics, frequency distributions, percentages, means, and standard deviations, and then subsequently executed the independent *t*-test, Pearson’s product-moment correlation coefficient analysis, and Multiple linear regression analysis used to examine the predictors for the KAP-PCCSI of healthcare members. The minimum sample size was estimated by G-power 3.1, while the *F* statistic for linear multiple regression set the parameters as below: statistical power was 0.8, significant level of alpha was 0.05, the number of predictors was four, and effect size value of *R*^*2*^ was 0.15 (medium). According to these parameters, the minimum sample size was estimated as 85.

### Ethics statement

This study was approved by the Kaohsiung Armed Forces General Hospital Institutional Review Board (KAFGH-106-040).

## Results

### Sociodemographic characteristics and their relatives

We enrolled 210 participants from a regional teaching hospital. The characteristics of these participants are listed in Table 1. The participants’ average age was 37.9 ± 8.9 years, and most of them were women (82.9%), with only a small portion being men (17.1%). Most of the participants had a bachelor’s degree (79.5%), and 59.0% of the participants were married. In addition, the participants’ average years of work experience in the current hospital were 11.7 ± 9.1 years, and their total years of work experience were 13.7 ± 8.7 years. Regarding religious beliefs, 32.4% of the participants reported Buddhism as their religion, and 32.4% reported having no religious beliefs. Nearly all participants (97.1%) had experienced the death of a family member(s) or friend(s), and most had experienced a patient expiring while they were on duty (89%). Many of the participants (71.9%) had taken PCCS courses, and slightly more than half of the participants (58.1%) reported having contact with the PCCS team five or more times in the past (Table 1).

**Table 1:** Sociodemographic data distribution.

Variable	All participants (n = 210)	Group1 (nurses) (n = 140)	Group2 (physicians and nurse practitioners) (n = 39)	Group3 (medical personnel) (n = 31)
**Age**^**a**^ **(y)**	37.9 ± 8.9	36.8 ± 9.2	39.8 ± 0.5	40.6 ± 8.8
**Sex, n (%)**				
Female	174 (82.9)	136 (97.1)	17 (43.6)	21 (67.7)
Male	36 (17.1)	4 (2.9)	22 (56.4)	10 (32.3)
**Education level, n (%)**				
Junior college	41 (19.5)	37 (26.4)	0	4 (12.9)
Bachelor’s degree	167 (79.5)	103 (73.6)	38 (97.4)	26 (83.9)
Master’s degree or above	2 (1.0)	0	1 (2.6)	1 (3.2)
**Marital status, n (%)**				
Unmarried	80 (38.1)	66 (47.1)	7 (17.9)	7 (22.6)
Married	124 (59.0)	70 (50.0)	30 (76.9)	24 (77.4)
Divorced	6 (2.9)	4 (2.9)	2 (5.1)	0
**Work experience in current hospital** ^**a**^ **(y)**	11.7 ± 9.1	12.4 ± 9.3	9.7 ± 8.3	10.8 ± 8.9
**Total work experience** ^**a**^ **(y)**	13.7 ± 8.7	13.6 ± 9.2	13.8 ± 6.5	14.6 ± 8.6
**Religious belief, n (%)**				
Buddhism & Taoism	115 (54.8)	75 (53.6)	23 (59.0)	17 (54.8)
Catholicism & Christianity	14 (6.7)	6 (4.3)	5 (12.8)	3 (9.7)
Others	13 (6.2)	10 (7.1)	3 (7.7)	0
No religious belief	68 (32.4)	49 (35.0)	8 (20.5)	11 (35.5)
**Experienced a family member(s) or friend(s) passed away, n (%)**				
Yes	204 (97.1)	136 (95.7)	39 (100)	31 (100)
No	6 (2.9)	6 (4.3)	0	0
**Experienced a patient expiring when on duty, n (%)**				
Yes	187 (89.0)	134 (95.7)	36 (92.3)	17 (54.8)
No	23 (11.0)	6 (4.3)	3 (7.7)	14 (45.2)
**Attended PCCS courses, n (%)**				
Yes	151 (71.9)	107 (76.4)	34 (87.2)	10 (32.3)
No	29 (28.1)	33 (23.6)	5 (12.8)	21 (67.7)
**Frequency of contact with PCCS team, n (%)**				
0 time	22 (10.5)	6 (4.3)	0	16 (51.6)
1~45 times	66(31.4)	41(29.3)	19(48.7)	6(19.4)
5 times or more	122 (58.1)	93 (66.4)	20 (51.3)	9 (29.0)

^a^ Mean ± SD

Group 1 was comprised of 140 nurses, with an average age of 36.8 ± 9.2 years, nearly all of them being women (97.1%) with only 2.9% being men. Most of the nurses held a bachelor’s degree (73.6%) and half of them were married (50%). Their average years of work experience in the current hospital were 12.4 ± 9.3 years, and the total years of work experience were 13.6 ± 9.2 years. The most common religious beliefs were Buddhism (29.3%) and no religion (35%). Approximately all of them (95.7%) had experienced the death of a family member(s) or friend(s), and nearly all of them (95.7%) had experienced a patient expiring while on duty. Most of the nurses had taken PCCS courses (76.4%), and more than half of them (66.4%) reported having contact with the PCCS team five or more times (Table 1).

Group 2 was comprised of 39 physicians and nurse practitioners, with average age being 39.8 ± 0.5 years and male and female proportions being 56.4% and 43.6% respectively. Nearly all physicians and nurse practitioners held a bachelor’s degree (97.4%), and most of them were married (76.9%).The average years of work experience in the current hospital were 9.7 ± 8.3 years, and the total years of work experience were 13.8 ± 6.5 years. Furthermore, Buddhism (33.3%) and Taoism (25.6%) were the most common religious beliefs. All physicians and nurse practitioners (100%) had experienced the death of a family member(s) or friend(s), and nearly all of them (92.3%) had experienced a patient expiring while on duty. Most physicians and nurse practitioners (87.2%) had taken PCCS courses, with approximately half of them (51.3%) having contact with the PCCS team five or more times (Table 1).

Group 3 was comprised of 31 medical personnel, with average age being 40.6 ± 8.8 years and male and female proportions being 32.3% and 67.7% respectively. Most of the medical personnel had a bachelor’s degree (83.9%), and most of them were married (77.4%).The average years of work experience in the current hospital were 10.8 ± 8.9 years, and the total years of work experience were 14.6 ± 8.6 years. Additionally, the most common religious beliefs were Buddhism (45.2%) and no religious beliefs (35.5%). All medical personnel (100%) had experienced the death of a family member(s) or friend(s), and approximately half of them (54.8%) had experienced a patient expiring while on duty. Most medical personnel (67.7%) had not taken the PCCS courses, with approximately half of the members (51.6%) having no contact with the PCCS team (Table 1).

### Knowledge, attitudes, and practices toward PCCS

The knowledge, attitudes, and practices toward PCCS are listed in Table 2. For all participants, the total score for knowledge practice towards PCCS was 58.7 ± 8.9 (perfect score: 75); that for attitude towards PCCS was 42.7 ± 4.7 (perfect score: 50), while that for practice toward PCCS was 36.3 ± 8.1(perfect score: 50).

**Table 2:** Knowledge, Attitudes, and Practices toward PCCS.

Variable	All participants (n = 210)	Group1 (nurses) (n = 140)	Group2 (physicians and nurse practitioners) (n = 39)	Group3 (medical personnel) (n = 31)
**Knowledge towards PCCS**	58.7 ± 8.9	59.5 ± 8.2	61.7 ± 6.7	51.5 ± 10.8
**Attitudes toward PCCS**	42.7 ± 4.7	43.2 ± 4.5	42.7 ± 4.2	40.6 ± 5.6
**Practices toward PCCS**	36.3 ± 8.1	36.9 ± 7.5	39.3 ± 5.6	29.9 ± 9.9

^a^ Mean ± SD

For group 1 (nurses), the total score for knowledge practice towards PCCS was 59.2 ± 8.2, that for attitude towards PCCS was 43.2 ± 4.5, while that for practice towards PCCS was 36.9 ± 4.5 (Table 2).

For group 2 (physicians and nurse practitioners), the total score for knowledge practice towards PCCS was 61.7 ± 6.7, that for attitude towards PCCS was 42.7 ± 4.2, while that for practice towards PCCS was 39.3 ± 5.6 (Table 2).

For group 3 (medical personnel), the total score for knowledge practice towards PCCS was 51.5 ± 10.8, that for attitude towards PCCS was 40.6 ± 5.6, while that for practice towards PCCS was 29.9 ± 9.9 (Table 2).

Correlation between knowledge, attitudes, and practices toward PCCS are listed in Table 3. Overall, positive correlations between all participants’ knowledge and practices toward PCCS (*r* = 0.57, *p* < 0.01) signified more knowledge towards PCCS engendered more favorable practices toward PCCS, while that between all participants’ attitudes and practices toward PCCS (*r* = 0.42, *p* < 0.01) suggested more positive attitudes toward PCCS induced more favorable practices toward PCCS; furthermore, that between all participants’ knowledge and attitudes toward PCCS (*r* = 0.54, *p* < 0.01) revealed more knowledge towards PCCS engendered more positive attitudes toward PCCS (Table 3).

**Table 3:** Correlational Analysis of Knowledge, Attitudes, and Practices toward PCCS.

Variable	Knowledge toward PCCS	Attitudes toward PCCS	Practices toward PCCS
**Knowledge toward PCCS**	1		
**Attitudes toward PCCS**	.54**	1	
**Practices toward PCCS**	.57**	.42**	1

*p < 0.05

**p < 0.01

### Predictors of practices toward PCCS

Predictors of practices toward PCCS are listed in Table 4. We conducted a multiple linear regression analysis to assess sociodemographic variables as well as knowledge, attitudes, and practices toward PCCS. We applied the “Enter Selection” method to select predictors. A test of collinearity showed no collinearity. The analysis results revealed that the significant predictors of practices toward PCCS were knowledge toward PCCS, attitudes toward PCCS, experience of a family member(s) or friend(s) passing away, and occupation category (F = 5.3, *p* < 0.001). Overall, these four predictors explained 43% of the variance in practices toward PCCS (△R^2^ = .43).

Every 1-point increase in knowledge toward PCCS was associated with a 0.3-point increase in practices toward PCCS; moreover, every 1-point increase in attitudes toward PCCS was associated with a 0.2-point increase in practices toward PCCS. An experience of a family member(s) or friend(s) passing away engendered a 6.4-point increase in practices toward PCCS, compared with those without such experiences. In the different occupation category, group 1 (nurses) outscored group 3 (medical personnel) by a 6.2-point increase in practices toward PCCS, whereas group 2 (physicians and nurse practitioners) outscored group 3 (medical personnel) by a 4.9-point increase in practices toward PCCS (Table 4).

**Table 4:** Multiple Linear Regression for Practices toward PCCS.

Variable	B	β	95% CI	Tolerance	VIF	R^2^	F value
**Constant**	-5.6		-24.0 to 12.8			.43	5.3***
**Knowledge towards PCCS**	.3	.3	.2 to .4***	.59	1.6		
**Attitudes toward PCCS**	.2	.1	.0 to .4*	.68	1.4		
**Occupation category**							
Group 3 (medical personnel)	Reference						
Group 1 (nurses)	6.2	.3	2.5 to 9.9**	.25	3.8		
Group 2 (physicians and nurse practitioners)	4.9	.2	.9 to 8.9*	.28	3.5		
**Experienced loss of a family member(s) or friend(s) passing away**							
No	Reference						
Yes	6.4	.1	.9 to 12.0*	.76	1.3		

CI = confidence interval

*p<0.05

** *p<*0.01

*** *p<*0.001

## Discussion

In this study, we determined that knowledge towards PCCS, attitudes toward PCCS, experience of the death of a family member(s) or friend(s), and being nurses, physicians or nurse practitioners were main predictors of practices toward PCCS. We also found that healthcare members’ knowledge and attitudes toward PCCS were positively correlated with their practices toward PCCS, signifying that these are positive predictors of the palliative care consultation service (PCCS). Most recent studies have suggested that knowledge towards PCCS of nurses is a major factor affecting their PCCS [16–18]. For physicians and nurse practitioners in healthcare members, knowledge and attitudes toward PCCS influence their willingness to cooperate with PCCS teams [19–22].

Accordingly, for healthcare members, PCCS training could not only enhance members’ understanding of palliative care but improve the quality of care for patients with terminal illness as well, thus improving the patients’ quality of life [23]. Previous research has indicated that knowledge towards PCCS of nursing staff members is correlated with their attitudes and coping skills practices when caring for patients with terminal illness [24]; furthermore, nursing staff members’ attitudes and coping practices are correlated with their level of preparedness for practicing palliative care.

A positive attitude toward PCCS of nursing staff promotes the referral of patients to the PCCS teams and provision of PCCS [25]. Previous research has also suggested that knowledge and attitudes toward palliative care constitute major barriers to the promotion of PCCS for the physicians and specialist physicians; therefore, research has recommended the implementation of systematic education programs specifically a series of education and training programs for palliative care should be implemented from the medical student stage through to the clinical stage. This increases the understanding of PCCS and willingness to cooperate with PCCS teams, thereby facilitating the establishment of a consensus between teams and promoting the application of clinical palliative care [21].

The PCCS primarily involves alleviating pain, managing symptoms, seeking spiritual health, and improving end-of-life quality. Occasionally, in the event of disagreements between family members, family meetings are held for determining appropriate care models. When primary health professionals have more knowledge about PCCS, they are more comfortable in providing information, recommendations, and better palliative care to patients and their family members [9,15,21,26–28], so promoting PCCS can considerably benefit patients and their family members thus enabling them to gain supportive and comprehensive care.

Regarding the promotion of palliative care in medical care systems, experience of losing a family member(s) or a friend(s) and contact with palliative care can influence the provision of PCCS [14,15,27–32]. However, numerous studies have reported that for patients with terminal illness and those with an unfavorable prognosis, nurses and physicians influence such patients’ and their family members’ acceptance of PCCS. Another factor promoting PCCS is a hospital’s marketing strategy for increasing the understanding of PCCS among patients [21]. The PCCS improves care quality, patient and family satisfaction, as well as hospital discharge rate [31,33–35]; therefore, the healthcare team members’ knowledge and attitudes toward PCCS affect their clinical care practices for patients with terminal illness.

### Limitations of the study

This study has some limitations. Firstly, the overall scale for assessing knowledge, attitudes, and practice toward PCCS is a self-reported questionnaire. Specifically, healthcare team members might be reluctant to express their true opinions because of concerns that their opinions might differ from those of their colleagues. Secondly, we adopted convenience sampling, and were restricted to a single regional teaching hospital; therefore, this study has limited generalizability. Caution should be exercised therefore in extrapolating the results. Thirdly, we applied a cross-sectional design for this study, and did not examine any changes in the knowledge, attitudes, and practices toward PCCS over time.

## Conclusions

The study determined that knowledge toward PCCS, attitudes toward PCCS, experience of the death of a family member(s) or friend(s) and being nurses, physicians or nurse practitioners constituted main predictors of healthcare team members’ practices toward PCCS. Accordingly, it is suggested that annual clinical education plans incorporate in-service education on palliative care in order to enhance medical teams’ positive attitudes toward PCCS, thereby promoting the provision of palliative care to patients with terminal illness, improving their quality of life and treatment satisfaction near the end of their life, as well as providing emotional support to their family members.

## Supporting information

S1 File Study’s minimal underlying data set. (XLSX)
